# Detection of glioma infiltration at the tumor margin using quantitative stimulated Raman scattering histology

**DOI:** 10.1038/s41598-021-91648-8

**Published:** 2021-06-09

**Authors:** Melike Pekmezci, Ramin A. Morshed, Pranathi Chunduru, Balaji Pandian, Jacob Young, Javier E. Villanueva-Meyer, Tarik Tihan, Emily A. Sloan, Manish K. Aghi, Annette M. Molinaro, Mitchel S. Berger, Shawn L. Hervey-Jumper

**Affiliations:** 1 Department of Pathology, University of California, San Francisco, CA USA; 2 Department of Neurological Surgery, University of California, 505 Parnassus Ave., Rm. M-779, San Francisco, CA 94143-0112 USA; 3 Department of Epidemiology and Biostatistics, University of California, San Francisco, CA USA; 4 Department of Radiology, University of California, San Francisco, CA USA; 5 Invenio Imaging, Inc, Santa Clara, CA USA

**Keywords:** Medical imaging, Brain imaging

## Abstract

In the management of diffuse gliomas, the identification and removal of tumor at the infiltrative margin remains a central challenge. Prior work has demonstrated that fluorescence labeling tools and radiographic imaging are useful surgical adjuvants with macroscopic resolution. However, they lose sensitivity at the tumor margin and have limited clinical utility for lower grade histologies. Fiber-laser based stimulated Raman histology (SRH) is an optical imaging technique that provides microscopic tissue characterization of unprocessed tissues. It remains unknown whether SRH of tissues taken from the infiltrative glioma margin will identify microscopic residual disease. Here we acquired glioma margin specimens for SRH, histology, and tumor specific tissue characterization. Generalized linear mixed models were used to evaluate agreement. We find that SRH identified residual tumor in 82 of 167 margin specimens (49%), compared to IHC confirming residual tumor in 72 of 128 samples (56%), and H&E confirming residual tumor in 82 of 169 samples (49%). Intraobserver agreements between all 3 modalities were confirmed. These data demonstrate that SRH detects residual microscopic tumor at the infiltrative glioma margin and may be a promising tool to enhance extent of resection.

## Introduction

Maximal safe extent of tumor removal is associated with improved overall and progression free survival for both high and lower grade infiltrating gliomas (grades II-IV per the World Health Organization (WHO) Classification of Central Nervous System tumors)^1–4^. Gliomas however exist within the context of complex neural networks contributing to neurological functions. Therefore, differentiating neoplastic tumor tissues from normal brain poses a challenge, especially at the infiltrative tumor margin, and may lead to suboptimal extent of resection^5^. Patterns of disease recurrence demonstrate that residual tumor near the resection cavity is the most common site of tumor recurrence^6^. Thus, techniques to identify microscopic disease intraoperatively at the tumor margin may help address suboptimal resection and improve patient outcomes.

Strategies have been developed to improve extent of glioma resection. Intraoperative frameless stereotactic navigation can identify a tumor border based on preoperative MRI. However, brain shift throughout the resection leads to inaccuracy in over 70% of cases^7^. Intraoperative MRI and fluorescence-guided surgery are other modalities used to aid in the intraoperative identification of residual tumor tissue and can improve extent of resection^3,8^. However, both techniques offer macroscopic visibility only. Furthermore, fluorescence guided surgery using techniques such as 5-aminolevulinic acid are most accurate when targeting contrast-enhancing disease with high cellularity. Yet, maximal resection of non-enhancing tumor tissue is the surgical goal for many patients^4^. Microscopic residual disease can be difficult to assess, regardless of the surgical adjunct used. Currently, intraoperative identification of tumor cells on a microscopic level can be achieved by frozen section assessment of the margins. This is a time- and labor-intensive process which is not feasible for rapidly detecting glioma cells within the tumor margin, especially when a resection cavity may be large and multiple areas must be sampled. Thus, rapid intraoperative serial specimen processing and histologic assessment is required in order to incorporate real-time microscopic assessment of glioma margins for consideration during cytoreductive surgery.

Stimulated Raman scattering histology (SRH) is a non-destructive, rapid, label-free technique that provides imaging of unprocessed surgical tissues at microscopic resolutions^9^. It relies on the Raman effect, which occurs when light temporarily changes a bond’s polarizability and causes a change in the vibrational frequency leading to a change in the energy of the scattered photon. This information can be displayed as a pseudo-histologic image, and previous studies showed that SRH images can be used to distinguish normal cortex, gliosis, and intrinsic and extrinsic CNS tumors similar to routine hematoxylin and eosin (H&E)-stained sections^10,11^. However, in order for SRH to be clinically useful for the evaluation of glioma margins, SRH results should correlate with standard techniques, not only at the tumor’s core, but also at the margins where cellularity and tumor percentage are expected to be lower. While the analysis of postmortem tissues has suggested the ability of SRH to detect glioma infiltration with high sensitivity and specificity^10,11^, there are no data assessing SRH within the operating room setting to quantify infiltrating tumor cells at the glioma resection cavity margins.

In this study, we examined glioma samples obtained from infiltrative tumor margins in order to determine whether SRH may identify microscopic residual disease. We subsequently processed the tissue samples using standard histologic processing and correlated the SRH results with H&E- and immunohistochemistry (IHC)-stained samples.

## Results

### Patient population and histopathology

There were 31 patients in the study with age at the time of surgery ranging from 22 to 83 years: 18 patients (58%) had glioblastoma WHO grade IV, 2 of which were IDH-mutant; 10 patients (32%) had IDH-mutant and 1p/19q-codeleted oligodendroglioma; and 3 patients had IDH-mutant anaplastic astrocytomas (Table 1). Additional information for tumors with positive and negative margins, including MRI characteristics, are summarized in Supplementary Table S1.

**Table 1:** Patient characteristics.

Characteristic	All (n = 31)
**Age**	
Median (range)	60 (22–83)
**Sex**	
Male (%)	17 (55)
Female (%)	14 (45)
**Diagnosis**	
Glioblastoma, IDH-wildtype, WHO grade IV*	16
Glioblastoma, IDH mutant, WHO grade IV	2
Anaplastic astrocytoma, IDH mutant, WHO grade III	3
Anaplastic Oligodendroglioma, IDH-mutant and 1p/19q-codeleted, WHO grade III	4
Diffuse astrocytoma, IDH mutant, WHO grade II	0
Oligodendroglioma, IDH-mutant and 1p/19q-codeleted, WHO grade II	6

*Two patients had IDH-wildtype astrocytomas with histologic grade of III; however, these were considered molecular glioblastoma using the cIMPACT-NOW criteria and included in the study as such^12^.

### Stimulated Raman microscopy of the infiltrative glioma margin

**Figure 1:**
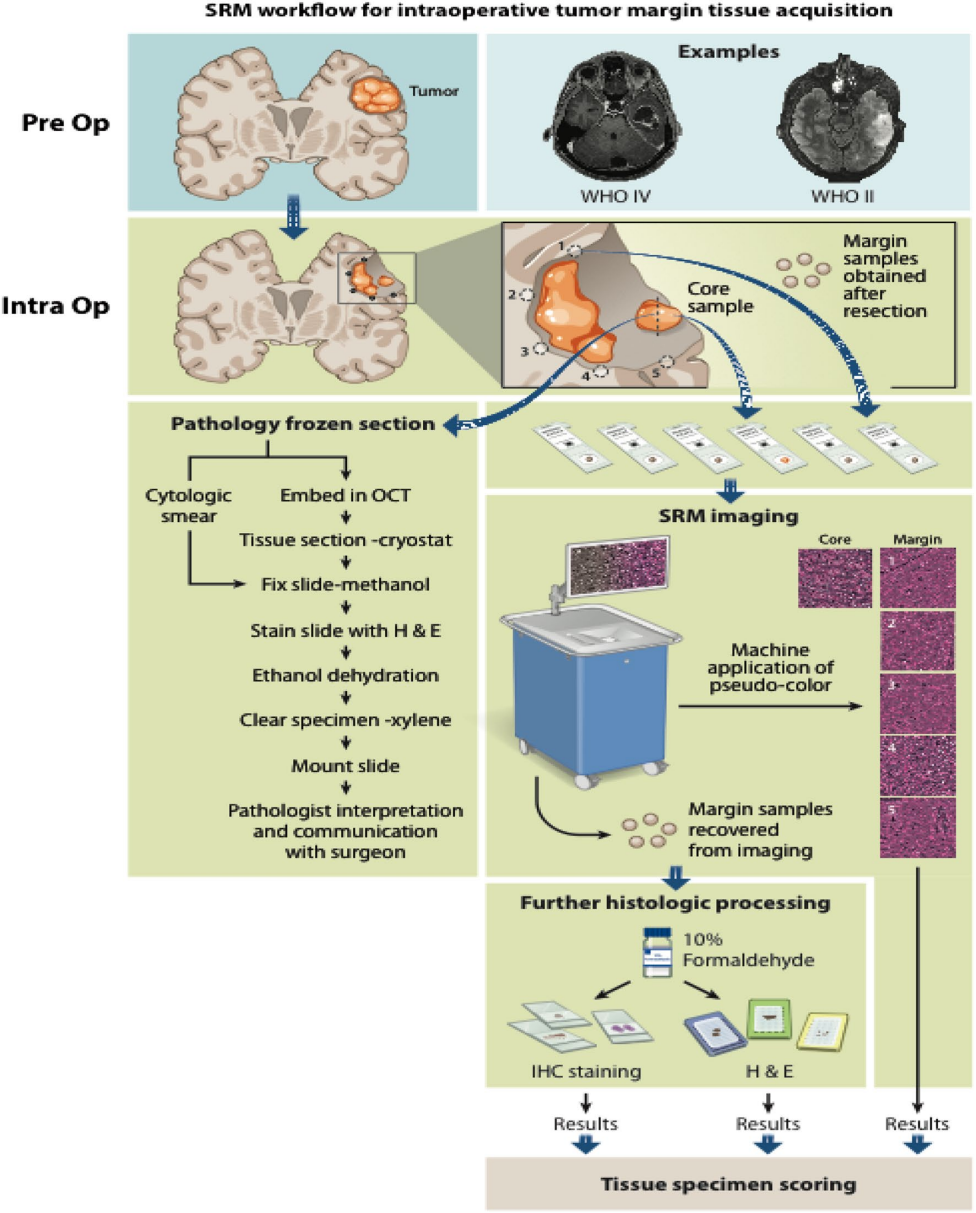
Stimulated Raman scattering histology (SRH) workflow for intraoperative tumor margin tissue assessment. Tumor samples obtained from the lesion core were divided for routine intraoperative evaluation using cytologic smear preparations and/or frozen section, and the remaining tissue was submitted for SRH imaging. Upon completion of resection, samples obtained from the resection cavity margins are submitted for SRH imaging. SRH images were obtained, and pseudo-H&E images were rendered. Tissues used for SRH are subsequently placed in 10% formaldehyde and submitted for histologic processing, paraffin embedding, sectioning, and staining. SRH images and histology sections from margins were retrospectively reviewed.

**Figure 2:**
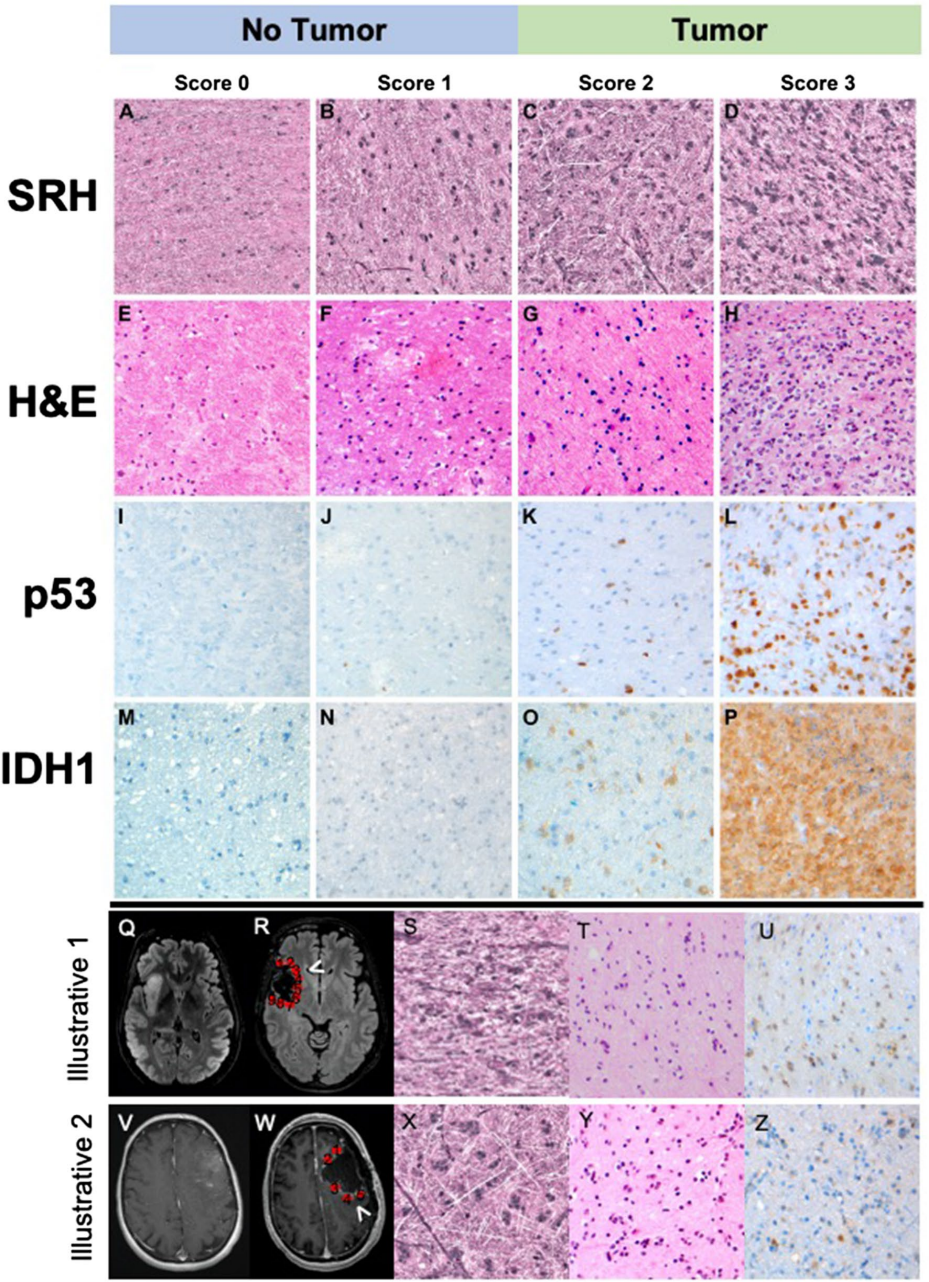
Representative images of SRH microscopy, and hematoxylin & eosin, IDH1 R132H and p53 stained slides corresponding to semiquantitative scores. Panels from right to left correspond to scores 0 through 3 for all modalities. Tumor margin scores: 0—no tumor; 1—rare atypical tumor cells, no definite tumor; 2—sparse tumor cells; 3—abundant tumor cells. (**A**–**D**) are stimulated Raman scattering (SRH) microscopy images after conversion of pseudo-H&E images. Panels (**E**–**H**) are photographs obtained from H&E-stained histology sections corresponding to the margins depicted in panels (**A**–**D**). Panels (**I**–**L**) are p53-stained sections, corresponding to the margins depicted in panels (**A**–**D**). Panels (**M**–**P**) are IDH1 R132 stained sections of different margins with similar scores. Two case illustrations of residual tumor at radiographically clean margins are provided. (**Q**–**U**) Oligodendroglioma, IDH-mutant, WHO grade II. Panels (**Q** and **R**) are T2 FLAIR MR images before and after surgery with red circles on (**B**) highlighting regions of margin sampling. (**S**, **T**, and **U**) are images from margin 3 using SRH, H&E and IDH1 R132H IHC, respectively. (**V**–**Z**) Glioblastoma, IDH-mutant, WHO grade IV. (**V** and **W**) are post contrast T1-weighted MR images before and after surgery with red circles on G highlighting regions of margin sampling. (**X**–**Z**) are images from margin 5 using SRH, H&E and IDH1 R132H IHC, respectively.

Prior published work has illustrated the ability of SRH to image fresh surgical specimens, revealing diagnostic features sufficient for the classification of CNS tumors including low- and high-grade glioma. The ultimate goal of this manuscript was to use SRH to image glioma margins, which are inherently less cellular than tumor core, for the purposes of identifying microscopic residual tumor (Fig. 1)^10,11,13,14^. Similar to conventional H&E sections, SRH images of low- and high-grade glioma margins revealed cellular and architectural differentiation permitting quantitative and semiquantitative assessments of tissue cellularity (Fig. 2A–D). Each tumor margin section was then sectioned for H&E (Fig. 1E–H), and IHC using either p53 (Fig. 1I–L) or IDH1 (Fig. 1M–P), with each sample scored for the presence or absence of tumor. Representative images of SRH and H&E-, IDH1 R132H- and p53-stained sections for this 2-tiered system are presented in Fig. 1A–P. A separate 4-tier scoring system was also developed to provide for a more detailed description of the degree of infiltration as well as histological nuances such as cellular atypia (4 tiered results shared in supplemental tables and figures).

### Stimulated Raman microscopy identified residual glioma at the margin

Gliomas have a propensity to infiltrate into brain parenchyma, which contributes to the high rate of local recurrence. During surgical removal, infiltrative glioma cells not visible by standard intraoperative techniques are known to extend beyond gross^15^ and radiographic^16^ margins of the tumor. We therefore set out to determine whether SRH might identify residual disease not evident by standard intraoperative techniques. All tumor margin samples were obtained at the point in which both intraoperative white-light microscope and neuro-navigation suggested gross-total removal of tumor with no macroscopic evidence of residual tumor. This resulted in 179 “margin” samples, all of which were acquired with stereotactic navigation coordinates. Post-operative MRI overlaid with navigation coordinates confirmed that 169 (94.4%) of these samples were indeed at the tumor margins as determined by T1 post gadolinium sequence for WHO IV gliomas or T2 FLAIR for WHO II-III gliomas, and used for the remainder of the analysis in the study. Ten of 179 (5.6%) samples were “within the tumor” per subsequent review of the imaging, and were not considered true margin samples. All 10 samples had residual tumor by IHC and SRH consensus scoring but were not included in the analysis.

**Figure 3:**
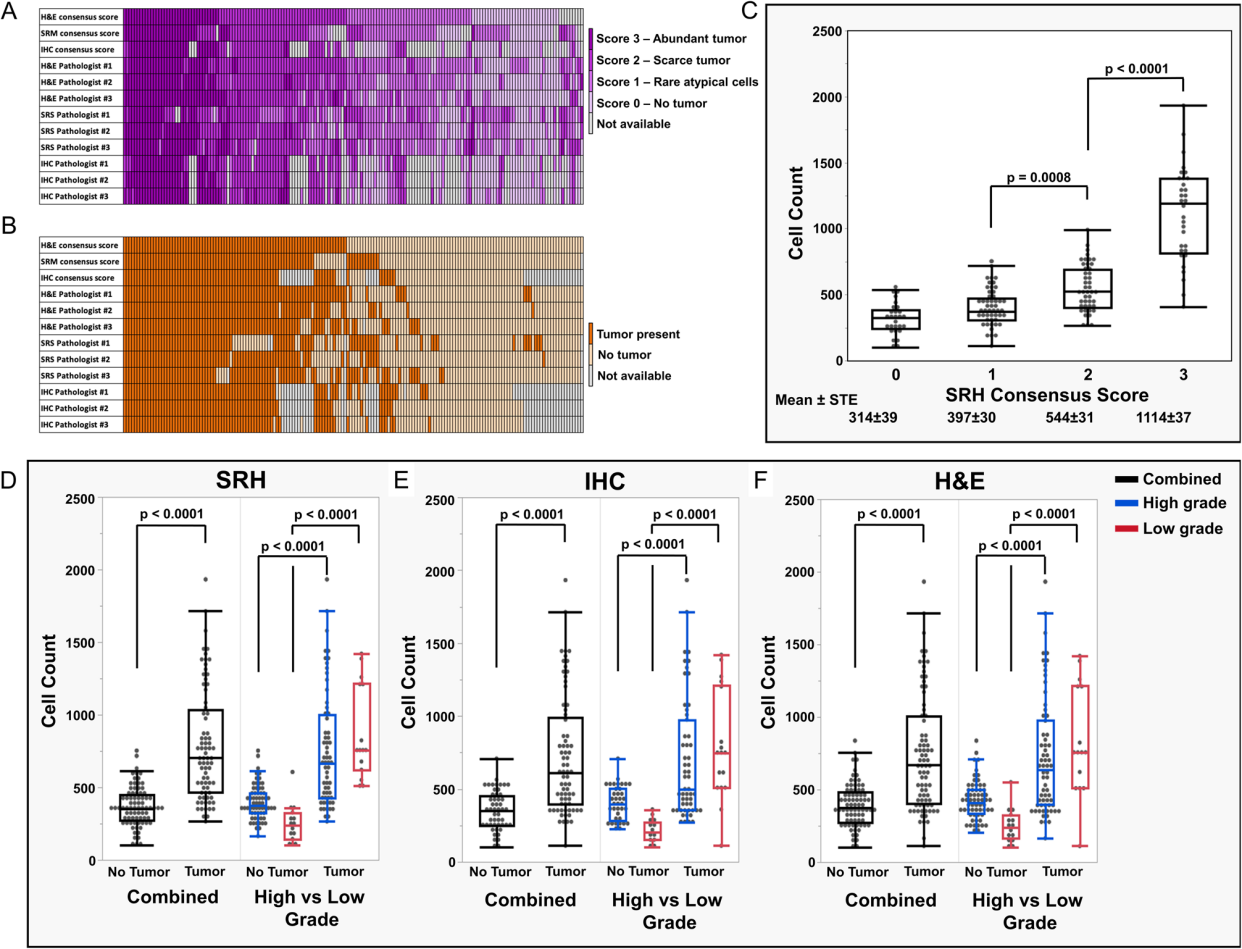
Heatmaps representing scores provided for each margin sample using Hematoxylin & Eosin (H&E) stained sections, Stimulated Raman Microscopy (SRH) images, and combined immunohistochemistry (IHC) for IDH1 R312H and p53. (**A**) Results of the 4-tier scoring system including consensus score and individual pathologist scores based on H&E, SRH, and IHC. (**B**) Results of the 2-tier, binary scoring system (tumor present vs. absent) including consensus score and individual pathologist scores based on H&E, SRH, and IHC. IHC score is not available for infiltrative gliomas which are IDH1 R132H-negative and p53-negative. Consensus score was rendered using the score provided by at least two pathologists for each modality for each margin. Consensus score was “not available” if pathologists provided three different scores. (**C**) Cellularity across different SRH consensus scores. There was a significant correlation between SRH consensus scores and cellularity of the tissue (*p* < 0.0001). Cell count (cell/mm^2^). (**D**–**F**) Samples with residual tumor demonstrated higher cellularity than samples without tumor for both low and high-grade gliomas, using a 2-tiered scoring system for all modalities. Panels (**D**–**F**) demonstrate the cellularity based on SRH, H&E and IHC (*p* < 0.0001).

Number of margins evaluated for each patient ranged from 1 to 12 (median 3). Presence of tumor was scored using a 2-tier system by all pathologists individually, and a consensus score among the pathologists for each sample using each modality. Results of the 2-tier scoring systems are summarized in Fig. 3A–C, respective.

We began with a 4-tier analysis of consensus scores. IHC (n = 122) samples demonstrated abundant tumor in 32 samples (26%), scarce tumor in 40 samples (33%), and rare atypical cells in 11 samples (9%), with 39 samples (32%) negative for tumor. Using the 4-tier consensus scores for H&E-stained sections (n = 160), there were abundant tumor in 35 samples (22%), scarce tumor in 47 samples (29%), and rare atypical cells in 46 samples (29%), with 32 samples (20%) negative for tumor. Analysis of tumor margin samples using the 4-tier consensus scores for SRH (n = 155) showed that 32 samples (21%) had abundant tumor, 45 samples (29%) had scarce tumor, 50 samples (32%) had rare, atypical cells, and 28 samples (18%) had no tumor.

Using the 2-tier scoring system, 72 of 128 samples (56%) with available consensus IHC score had residual tumor. Similarly using the 2-tier scoring system, 82 of 169 samples (49%) with available consensus H&E score, and 82 of 167 samples (49%) with available consensus SRH scores had residual tumor. Two illustrative examples with residual tumor at margin are provided in Fig. 2Q–Z.

When examining all samples within the cohort, the sensitivity for SRH to detect tumor by H&E and IHC was 0.86 (CI 0.77–0.93) and 0.88 (CI 0.76–0.95) respectively. The specificity for SRH to identify absence of tumor was 0.86 (CI 0.77- 0.93) for H&E and 0.81 (0.70- 0.89) for IHC. With respect to PPV, the probability that samples with substantial residual glioma (semiquantitative score 2–3) by SRH also have residual tumor (semiquantitative score 2–3) by H&E was 0.87 and IHC 0.78. The NPV for SRH with H&E was 0.85 and IHC was 0.89.

### Assessment of glioma margins using multiple modalities reveals agreement between SRH and H&E- and IHC-stained sections

We then set out to measure intraobserver agreements between the three modalities using the 2-tier consensus scores (Table 2A). Agreement between IHC and SRH was near perfect with Κ 0.84 (CI 0.750–0.94). There was also substantial agreement between IHC and H&E with Κ 0.67 (CI 0.54–0.80) and between SRH and H&E with Κ 0.72 (CI 0.62–0.83). We then set out to examine modality agreement based on WHO grade. Of the 16 imaged regions from WHO 2, low-grade lesions with tumor present (2-tier score 2–3), 13 slides had tumor present by SRH (13/16 or 81.3% accuracy by specimen; 7/7 = 100% accuracy by patient). For the 52 sections from WHO 3–4, high-grade lesions with tumor present, 42 slides had tumor by SRH (42/52 or 80.8% accuracy by specimen; 14/19 = 73.4% accuracy by patient). The Kappa agreement for SRH and IHC for low-grade gliomas was 0.74 (95% CI: 0.51–0.98) and for high-grade gliomas was 0.65 (95% CI: 0.5–0.9) (Table 2B).

**Table 2:** (A) Intraobserver agreement between modalities using a 2-tier semiquantitative consensus scores (B) Interobserver agreement between all three pathologists using a 2-tier semiquantitative scores for each modality.

	2-tier scoring system	
	Kappa	Lower–Upper CI
**A. Intraobserver agreement between modalities**		
SRH-H&E agreement	0.72	0.62–0.83
IHC-H&E agreement	0.67	0.54–0.80
IHC-SRH agreement	0.84	0.75–0.94
**B. WHO grade intraobserver agreement between modalities**		
Low grade: IHC-SRH agreement	0.74	0.51–0.98
High grade: IHC-SRH agreement	0.65	0.5–0.8
**C. Interobserver agreement between pathologists**		
SRH agreement	0.43	0.39–0.47
H&E agreement	0.60	0.56–0.63
IHC agreement	0.71	0.67–0.74

Interobserver agreement between all three pathologists varied based on modality. For IHC sections, there was substantial agreement as illustrated by Κ 0.71 (CI 0.67–0.74) (Table 2C). Kappa scores for H&E-stained and SRH images were 0.60 (CI 0.56–0.63) and 0.43 (CI 0.39–0.47), respectively (see supplemental Table 2 for intraobserver agreement between modalities and interobserver agreement between all three pathologists using 4-tier semiquantitative consensus scores).

We then evaluated the intraobserver agreements between modalities for each individual pathologist. Intraobserver agreements using 2-tier scoring system were near perfect for nearly all of the modalities with pathologist #2, who had the most experience with SRH (Table 3). Interobserver agreements were also calculated for each pathologist pair, and show significant variation based on the level of experience in neuropathology as well as experience with SRH (Table 4) (see supplemental Table 3 for 4-tier intraobserver agreement between modalities for each pathologist).

**Table 3:** Intraobserver agreement between modalities using 2-tier scoring systems for each pathologist.

	2-tier		
	(Pathologist #1)	(Pathologist #2)	(Pathologist #3)
SRH-H&E agreement	0.47 (0.34, 0.60)	0.80 (0.70, 0.89)	0.78 (0.69, 0.88)
IHC-H&E agreement	0.74 (0.62, 0.86)	0.89 (0.81, 0.97)	0.73 (0.61, 0.85)
IHC-SRH agreement	0.41 (0.25, 0.56)	0.77 (0.66, 0.88)	0.50 (0.34, 0.65)

**Table 4:** Interobserver agreement between pathologists using 2-tier scoring system for all modalities.

	All 3 pathologists	Pathologists #1 versus #2	Pathologists #1 versus #3	Pathologists #2 versus #3
SRH agreement	0.43 (0.39–0.47)	0.51 (0.38–0.64)	0.33 (0.19–0.47)	0.58 (0.46–0.70)
H&E agreement	0.60 (0.56–0.63)	0.75 (0.65–0.85)	0.69 (0.58–0.80)	0.65 (0.54–0.77)
IHC agreement	0.71 (0.67–0.74)	0.81 (0.71–0.92)	0.81 (0.70–0.92)	0.87 (0.79–0.96)

### Association between tissue cellularity and SRH score

In general, increased tissue cellularity correlates with the presence of tumor. However, lower grade diffuse gliomas and the infiltrating margins of high-grade gliomas may have cellularity similar to that of normal brain parenchyma. We therefore set out to determine the correlation between semiquantitative neuropathology scores and tissue cellularity of SRH imaged samples. Using semiquantitative consensus scoring (Fig. 2A,B) we correlated SRH imaged tissue cellularity with scoring for each modality. SRH cellularity was measured using an operator inspected machine learning technique outlined in Supplementary Figs. S1 and S2. Mean number of cells per mm^2^ for scores 0, 1, 2, and 3 respectively were 314 ± 39, 397 ± 30, 544 ± 31, and 1114 ± 37 (*p* < 0.0001) (Fig. 2C).

Using the 2-tier scoring system, margin samples with residual tumor (semiquantitative score 2–3) demonstrated greater cellularity than samples without tumor (semiquantitative score 0–1), across all three modalities for both low- and high-grade lesions (*p* < 0.0001 for all; Fig. 2D–F).

## Discussion

The identification of residual tumor at the infiltrative tumor margin remains a central challenge in glioma surgery, as residual tumor is the most common site of disease progression. Currently, microscopic analysis of tumor margins via intraoperative frozen sections is not routinely used to guide surgery, primarily due to the feedback time between tissue acquisition and actionable results^10^. Here, we demonstrate that SRH imaging of the glioma margins is able to generate pseudo-H&E images, which permits microscopic level detection of residual glioma cells, which were not identified by current intraoperative imaging methods including white-light microscopy and neuro-navigation. Furthermore, SRH has excellent agreement with p53 and IDH1 R132H IHC, which can be accepted as gold standard for microscopic tumor burden given their high sensitivity and specificity as surrogate markers for molecular alterations.

The use of a two pseudo-color SRH method (pseudo-H&E) has been described for the assessment of tissues from the tumor core^10,11,13,14^. SRH is based on the Raman spectral differences within the imaged tissue, which reflect variations in the lipid/protein ratio. These variations are used to generate contrast, which can be subsequently colored for visual inspection. In contrast to core tumor specimens, the utility of SRH for evaluation of presumably less cellular tumor margins has remained undetermined. Prior work has suggested the potential for SRH to reveal dense tumor, infiltrative tumor, and normal tissues in glioblastoma samples using cadaveric specimen imaged ex vivo^11^. Until now, this work has not been validated in the intraoperative setting. In addition to semiquantitative scoring of the margins by three neuropathologists using morphologic features, we have performed a cellularity count, which, as expected, correlates with the semiquantitative scoring models employed in this study. Nevertheless, the cellularity shows significant overlap between tissues with no tumor, rare atypical cells, and scarce tumor. The fact that SRH images can be used successfully to identify margins with scarce tumor confirms that SRH imaging provides sufficient cellular and architectural details, beyond cellularity. These features include the size and borders of the glial nuclei, perineuronal satellitosis, and presence of entrapped cortical neurons and entrapped axons, features similar to those neuropathologists use in routine diagnosis of H&E-stained slides. Pseudo-H&E SRH images resemble routine H&E in many ways, including basophilic (purple) nuclei, eosinophilic (pink) brain parenchyma and even darker pink glial process in reactive astrocytes or some gliomas^10^.

The infiltrative nature of gliomas has been well established^16,17^. With advances in glioma diagnosis and molecular techniques, reliable surrogate markers of tumor-defining molecular alterations can be used as the gold standard by which microscopic residual disease is determined. Therefore, multimodal tissue analysis comparing SRH with H&E and IHC techniques provided useful comparisons. IDH1 R132H antibody is highly sensitive and specific for this mutation, defining a large subset of lower grade diffuse gliomas, and is not seen in normal tissues^18^. Therefore, presence of any IDH1 R132H staining at the margin proves presence of residual tumor. On the other hand, p53 staining is usually a range in gliomas, and rare positive nuclei can also be present in normal tissues^19,20^ which may be prone to subjective interpretation. Nevertheless, interpretation of a stain as “positive” or “negative” is typically more objective and reproducible than evaluating the cytologic atypia or cellularity, which likely contributes to the high kappa values with near perfect interobserver agreement between pathologists, especially using a binary scoring system.

Our study included 179 tissue samples, of which 5.6% were not true margin samples, despite the use of gross inspection and neuro-navigation. Unsurprisingly, all non-margin specimens demonstrated abundant or scarce residual tumor using semiquantitative methods, suggesting the potential benefit of SRH to guide intraoperative decision making. The remaining specimens, all of which represent radiographically clean margins, unsurprisingly demonstrated residual tumor using IHC in 56% of samples. H&E and SRH showed residual microscopic tumor in 49% of samples, which is slightly less than our gold standard method using IHC, but a significant portion of the samples which were considered to be negative for tumor based on intraoperative white-light microscopy and neuro-navigation. This further supports the potential benefit of intraoperative SRH to assess margins. A 2-tier scoring system demonstrated agreement between the modalities as well as reproducibility among the pathologists. Considering the clinical applications and intraoperative use of margin assessments, a binary scoring system with a directly actionable result may be preferred, especially if it provides better reproducibility. However, a 4-tier scoring system can provide for greater tissue description.

Our results confirm that the tissue used for SRH can be subsequently submitted for routine histology without significant crushing or other processing artifacts. Furthermore, we showed that the tissue retains its immunogenic properties for subsequent IHC. It is generally accepted that frozen section remnants may show artifacts as well as variation in immunogenicity, supporting an additional benefit of SRH imaging over traditional frozen section techniques.

Limitations of this study design may impact interpretation of results. We elected to use the same tissue specimen for SRH, IHC, and H&E analysis. Duplicate use of the same tissue highlights a potential strength of SRH, in that imaged tissues may be used for subsequent analysis. However, we may have missed the subtle distortions in histology caused by SRH imaging. Furthermore, there is the potential for heterogeneity given that even marked SRH images tissues may still have some distortion following tissue sectioning for IHC and H&E. Next, SRH used in this context requires sampling of tissues outside of the operative field which may pose limitations in select settings. While we assess the role of SRH as a potential intraoperative technique, we have not compared its sensitivity and specificity with intraoperative frozen sections, because we believe IHC stains represent the gold standard and provide a more important comparison. Furthermore, pseudo-colored SRH images are similar to H&E, but not identical. There is therefore a learning curve for the successful interpretation of SRH images. Additional studies focusing on the role of pathologist experience can be considered for optimization of training. To decrease individual biases in this study, we used a consensus score, in which semiquantitative scores were assigned by at least 2 pathologists. Finally, our study design leaves unanswered whether SRH may enhance extent of glioma resection and whether greater extent of resection in this context will improve patient survival.

To summarize, in the surgical management of gliomas, the identification and removal of disease at the infiltrative margin remains a central challenge. Fluorescence labeling techniques and radiographic imaging are useful macroscopic measures of disease however they represent estimates tumor cellularity and loose sensitivity with lower grade histology. Intraoperative neuropathologic evaluation of resection cavity margins can be limited by time constraints and may not be feasible at many medical centers. Given that the majority of low- and high-grade gliomas recur within close proximity to the resection cavity margin, rapid intraoperative detection of residual glioma may improve patient outcome. In this study, we demonstrate that SRH, a rapid optical imaging method, can identify residual glioma within tissue samples from the infiltrative glioma margin. Quantification of SRH images correlate with tissue cellularity and have excellent agreement with H&E and immunohistochemistry stained samples. These results demonstrate how SRH may facilitate more complete glioma resections.

## Methods

### Patient selection and study design

In this prospective single-center study, inclusion criteria included: (1) adult subjects age 18 -85; (2) able to give informed consent; (3) with presumed WHO II-IV glioma based on preoperative T1 post gadolinium and T2 FLAIR MRI sequence; (4) scheduled for a craniotomy for brain tumor resection at the University of California, San Francisco; (5) in which there is brain tumor tissue at the tumor margins deemed safe for sampling by the attending neurosurgeon. The study was approved by the Human Research Protection Program Institutional Review Board (IRB) of the University of California San Francisco (UCSF). All patients gave their written informed consent for the scientific use of their data. The study was performed in accordance with the Declaration of Helsinki 2013 and based on the principles of the International Conference on Harmonization: Good Clinical Practice guidelines. Patient enrollment began January 2019 and ended May 2019. All samples were obtained by study surgeons (SHJ, MKA, and MSB) once they considered tumor margins to be free of gross residual tumor based on white light microscopy and neuro-navigation. Neither fluorescence guided surgery (5-aminolevulinic acid) or intraoperative MRI were used for patients during this study. All samples were retrospectively evaluated by all three neuropathologists (MP, EAS and TT) at the conclusion of the study, and no real-time input was provided to the surgeons at the time of surgery. All pre- and intraoperative images were reviewed by a neuro-radiologist (JV-M) to confirm that each sample was truly at the margins of T1 post gadolinium signal for WHO IV gliomas or T2 FLAIR signal for WHO II and III gliomas^21^. Demographic information, final pathologic diagnosis, and WHO grade were obtained from electronic medical records.

### Study workflow

A total of 179 margin samples were procured from 31 patients undergoing brain tumor resection and were included in the study (Fig. 3). Upon appropriate surgical resection of presumed infiltrating gliomas, study surgeons evaluated the extent of resection using white light microscopy and neuro-navigation. Following suspected gross total resection of the tumor, multiple biopsies were taken around the tumor margins and X,Y,Z coordinates determined using Brainlab neuro-navigation were recorded. All margin samples were acquired from regions deemed to be not eloquent based on standard of care surgical protocols such as intraoperative functional mapping functional neuro-navigation using diffusion tensor imaging (DTI) MRI and magnetoencephalography. Margin specimens were imaged fresh with SRH, inked for orientation and subsequently placed in formalin for routine processing. SRH images and tissue sections stained with H&E and IDH1 R132H or p53 IHC were reviewed and scored using a semiquantitative scoring system.

### SRH imaging

A clinical, fiber-laser-based, stimulated Raman scattering microscope (Invenio Imaging Inc, Santa Clara, CA), which was designed for use in the operating room, was used for the study^22^. The stimulated Raman microscopy imaging platform involved five major components: (1) a fiber-coupled microscope with a motorized stage; (2) a dual-wavelength fiber-laser module; (3) a laser control module; (4) a microscope control module; and (5) a computer for image acquisition, display and processing. The entire system is mounted in a portable, self-contained clinical cart and utilizes a standard wall plug. The methods for SRH have been previously described in detail^10,11,13,14^.

Briefly, the tissue is excited with a dual-wavelength fiber laser with a fixed wavelength pump beam at 790 nm and a Stokes beam tunable from 1015 to 1050 nm. This configuration allows for spectral access to Raman shifts in the range from 2800 to 3130 cm^−1^^11^. Within imaged tissues the Raman spectral differences reflect variations in the lipid/protein ratio, and this is used to generate contrast. Images are acquired via beam scanning with a spatial sampling of 450 nm pixel^−1^, 1,000 pixels per strip and an imaging speed for 0.4 Megapixels per Raman shift. A sliding window algorithm with 100-pixel step size (both horizontal and vertical directions) and valid padding was used to generate 300 × 300 μm^2^ field of view (FOV) image patches at a rate of two seconds per frame in a mosaic pattern which are then stitched together. SRM images for this study were collected in two Raman frequencies corresponding to CH_2_ bonds which are abundant in lipids (2845 cm^−1^) and CH_3_ which predominate in proteins and DNA (2,930 cm^-1^) for each. The 2845 cm^−1^ images were subtracted from the 2930 cm^−1^ image, and the resultant image was concatenated to generate a three-channel image (2930 cm^−1^ minus 2845 cm^−1^, red; 2845 cm^−1^, green; 2930 cm^−1^, blue). This would allow transformation of raw SRM images to pseudo-H&Es with colors similar to routine H&E stains. Prior work has demonstrated that the mean time from tissue to the mosaic pseudo H&E image is 2.5 min^11^. SRM images were reviewed using the integrated high-definition monitor, remotely via a cloud-based image viewer that allows images to be reviewed anywhere with a high-speed internet connection of less than 30 s.

### Routine histology and IHC

Upon completion of SRH imaging, the region of tissues imaged were marked with tissue ink for orientation purposes and placed in 10% formaldehyde solution. Following routine processing, tissues were embedded in paraffin in an orientation allowing the cut surface to be in the same plane as the SRH image, and 5-micron sections were cut and stained with H&E using an automated stainer. Diagnosis and classification of infiltrating gliomas routinely requires application of IHC stains as surrogate markers for molecular alterations. IDH1 R132H mutation specific antibody is a highly sensitive and specific surrogate marker for this mutation, and any positive cell at the margin samples would be consistent with residual tumor^18^. Alterations in *TP53* are associated with increased p53 expression, and while a definitive cut-off is not established, staining in 50% or more of the tumor cells shows significant correlation with mutation, which will be referred to as p53-positive thereafter. Presence of p53-positive cells at the resection margin would be highly suspicious, but not entirely specific for residual tumor^19^. For this study, results of the IDH1 R132H and p53 staining performed on tumor samples sent to the clinical pathology laboratory were obtained from the electronic database. All margin samples from tumors with positive IDH1 R132H were stained with IDH1 R132H (clone H09, Dianova GmbH, Hamburg, Germany) using standard techniques. For IDH1 R132H-negative tumors, all margin samples from p53-positive tumors were stained with p53 (clone DO-7, Dako, Agilent, Santa Clara, CA). IDH1 R132H- and p53-stained sections are expected to have high sensitivity and specificity for tumor detection and are considered gold-standard.

### Scoring of H&E, SRH, and IHC slides

All SRH images, H&E- and IHC-stained slides were reviewed in a blinded fashion by three neuropathologists (MP, EAS, TT) independently, in a random order and semi quantitatively scored as follows: 0—No tumor; 1—Rare atypical cells but no definite tumor; 2—Tumor cells present, scarce; and 3—Tumor cells present, abundant. Representative images of each score using all modalities are presented in Fig. 1. Neuropathologists were blinded to each other’s scores.

On H&E-stained sections and SRH images, tumor cells were identified based on their morphologic features (i.e. enlarged and/or irregular and hyperchromatic nucleus) and architectural distribution (i.e. perineuronal satellitosis). On IDH1 R132H-stained sections, any positive cell was considered as residual tumor and scored as 2 or 3 based on the amount. Rare cases with minimal blush-like staining were scored as 1. On p53-stained sections, presence of numerous positive nuclei was scored as 3 (abundant tumor), and presence of scattered positive cells were scored as 1 or 2, based on the nuclear morphology and distribution. IHC score was provided combining IDH1 R132H and p53 results since each margin has only one of these stains performed. Immunohistochemical stains uninterpretable due to technical problems, such as tissue falling off the slide or failed stains, are not scored. Margins from IDH1 R132H-negative and p53-negative tumors do not have an IHC score.

In addition to the 4-tier system described above, a binary result for presence or absence of residual tumor at the margin was also provided combining scores 0 and 1 as “negative margin” and scores 2 and 3 as “positive margin” for all modalities. Finally, a consensus score among the pathologists was rendered using agreement by at least two pathologists for each modality for each margin. Consensus score was “not available” if pathologists provided three different scores.

### Cell counts

SRH microscopy images were segmented into cells using custom software developed in C++ 2017 utilizing the OpenCV library (version 4.1.2). The segmentation algorithm first subtracted the field-flattened CH2 channel (2845 cm^−1^) from the field-flattened CH3 channel (2930 cm^-1^). A binary mask was then applied such that pixels smaller than a specified threshold are zeroed. Next, a sequence of morphological erosion, morphological dilation, and morphological erosion filters were applied to the binary image. Remaining contiguous binary blobs are excluded by any of the following 3 metrics: (1) pixel size < 105; (2) pixel size > 1500; and (3) circularity score < 0.3. Circularity score for a given blob was defined as (4.0 * Pi * blob_area)/(perimeter * perimeter). Blobs that satisfied all of these filters were considered cells and counted. All SRH images with a consensus score were evaluated, and the cell counts were provided as cells per mm^2^.

### Statistical methods

The interobserver (measure of agreement between pathologists using each modality) and intraobserver (measure of agreement between modalities for each pathologist or the consensus score) agreements were calculated using Cohen’s kappa. Perfect agreement is indicated by a kappa value of 1.0, whereas a kappa value of 0 indicates no agreement at all. Previous studies used the following cut-offs to define the level of agreement: < 0.20, slight agreement; 0.21–0.41,fair agreement; 0.41–0.60, moderate agreement;0.61–0.80, substantial agreement; and 0.81–0.99, almost perfect agreement between observers^23^. Since Cohen’s kappa statistic is designed for measuring the agreement between two raters, ordinal generalized linear mixed models (GLMMs) were used to generate model‐based kappa statistics for measure of agreement across three pathologists for different modalities (SRH, H&E, and IHC) (See Tables 3, 4)^24^. The diagnostic accuracy of the binary score (negative or positive for residual tumor) was assessed using a receiver operating characteristic (ROC) curve. Previous studies used the following cut-offs to define accuracy: 0.5–0.6, fail; 0.6–0.7, poor accuracy; 0.7–0.8, fair accuracy; 0.8–0.9, good accuracy; and 0.9–1.0, excellent accuracy. Distribution of cell counts were compared across consensus SRH scores using 2-way ANOVA test. Sensitivity, specificity, positive predictive value (PPV), and negative predictive value (NPV) were calculated and reported with confidence intervals using 2-tiered semiquantitative scoring. All analyses were conducted using the statistical software R (http://www.r-project.org/).

## Supplementary Information

Supplementary Information.
