# Supplementary material for: Detection of glioma infiltration at the tumor margin using quantitative stimulated Raman scattering histology
Source: Sci Rep. 2021 Jun 9;11:12162. doi: 10.1038/s41598-021-91648-8 (PMC8190264; doi:10.1038/s41598-021-91648-8)

**Detection of glioma infiltration at the tumor margin using quantitative stimulated Raman scattering histology**

Melike Pekmezci MD^1^, Ramin A. Morshed MD^2^, Pranathi Chunduru MS^2, 3^ , Balaji Pandian BA^2, 5^, Jacob Young MD^2^, Javier E. Villanueva-Meyer MD^4^, Tarik Tihan MD PhD^1^, Emily A. Sloan MD PhD^1^, Manish K. Aghi MD PhD^2^, Annette M. Molinaro PhD^2,3^, Mitchel S. Berger MD^2^, Shawn L. Hervey-Jumper MD^2^

**Supplementary Material**

**Supplementary Table S1:** Detailed Patient Characteristics

**Supplemental Table S2:** Intraobserver agreement between modalities using a 4-tier semiquantitative consensus scores. (B) Interobserver agreement between all three pathologists using a 4-tier semiquantitative scores for each modality

**Supplemental Table S3:** Intraobserver agreement between modalities using 4-tier semiquantitative scores for each pathologist.

**Supplementary Figure S1:** Cell Counting Quantification Troubleshooting

**Supplementary Figure S2:** SRH Cellularity

**Supplementary Table S1: Detailed Patient Characteristics**

| Subject # | # of cores | # of margins | Positive margins by SRH | Negative margins by SRH | Sex | Age | Histo-logic WHO grade | IDH-mutant | Oligoden-droglioma, grade 2 or 3 | Astro-cytoma, grade 2 or 3 | Molecular GBM, grade 4 | Location | T1 enhancement | T2 FLAIR only (no  enhancement) |
| --- | --- | --- | --- | --- | --- | --- | --- | --- | --- | --- | --- | --- | --- | --- |
| 1 | 1 | 4 | 1 | 3 | male | 73 | 4 | no | no | no | no | temporal | yes | no |
| 2 | 1 | 4 | 2 | 2 | male | 22 | 3 | yes | no | yes | no | insula | no | yes |
| 3 | 1 | 2 | 0 | 2 | male | 72 | 4 | no | no | no | no | temporal | yes | no |
| 4 | 1 | 7 | 5 | 2 | male | 44 | 3 | yes | no | yes | no | temporal | no | yes |
| 5 | 1 | 2 | 0 | 2 | male | 53 | 4 | no | no | no | no | frontal | yes | no |
| 6 | 1 | 2 | 0 | 2 | male | 56 | 3 | yes | yes | no | no | frontal | yes | no |
| 7 | 1 | 8* | 6 | 1 | female | 82 | 3 | yes | yes | no | no | frontal | no | yes |
| 8 | 1 | 12 | 1 | 11 | male | 53 | 4 | no | no | no | no | frontal | yes | no |
| 9 | 1 | 2 | 0 | 2 | male | 48 | 4 | no | no | no | no | parietal | yes | no |
| 10 | 1 | 2 | 2 | 0 | female | 70 | 2 | yes | yes | no | no | frontal | no | yes |
| 11 | 1 | 11* | 3 | 7 | female | 63 | 2 | yes | yes | no | no | insula | no | yes |
| 12 | 1 | 1 | 0 | 1 | male | 68 | 4 | no | no | no | no | frontal | yes | no |
| 13 | 1 | 2 | 2 | 0 | male | 55 | 3^ | no | no | no | yes | insula | no | yes |
| 14 | 1 | 6 | 6 | 0 | female | 28 | 4 | no | no | no | no | parietal | no | yes |
| 15 | 1 | 9 | 1 | 8 | female | 61 | 3 | yes | yes | no | no | frontal | yes | no |
| 16 | 1 | 12 | 1 | 11 | female | 30 | 2 | yes | yes | no | no | frontal | no | yes |
| 17 | 1 | 5 | 1 | 4 | male | 75 | 3^ | no | no | no | yes | temporal | yes | no |
| 18 | 1 | 7 | 2 | 5 | female | 73 | 4 | no | no | no | no | temporal | yes | no |
| 19 | 1 | 2 | 1 | 1 | male | 83 | 4 | no | no | no | no | temporal | yes | no |
| 20 | 1 | 11 | 4 | 7 | female | 55 | 3 | yes | yes | no | no | frontal | no | yes |
| 21 | 1 | 6 | 6 | 0 | female | 68 | 4 | yes | no | no | no | frontal | yes | no |
| 22 | 1 | 2 | 2 | 0 | female | 45 | 4 | no | no | no | no | frontal | yes | no |
| 23 | 1 | 2 | 1 | 1 | male | 42 | 2 | yes | yes | no | no | occipital | no | yes |
| 24 | 1 | 4 | 4 | 0 | male | 63 | 4 | yes | no | no | no | insula | yes | no |
| 25 | 1 | 8 | 2 | 6 | male | 77 | 4 | no | no | no | no | parietal | yes | no |
| 26 | 1 | 7 | 6 | 1 | female | 47 | 4 | no | no | no | no | temporal | yes | no |
| 27 | 1 | 11 | 7 | 4 | male | 57 | 4 | no | no | no | no | parietal | yes | no |
| 28 | 1 | 8 | 6 | 2 | female | 60 | 3 | yes | no | yes | no | temporal | yes | no |
| 29 | 1 | 2 | 2 | 0 | male | 68 | 4 | no | no | no | no | frontal | yes | no |
| 30 | 1 | 2 | 2 | 0 | female | 39 | 2 | yes | yes | no | no | frontal | no | yes |
| 31 | 1 | 6 | 6 | 0 | female | 67 | 2 | yes | yes | no | no | parietal | no | yes |

* no SRH consensus neuropathology score

^ Astrocytomas with histologic grade of III harboring molecular alterations of glioblastoma, and therefore classified as molecular glioblastoma^10^

Supplemental Table 2. **(A) Intraobserver agreement between modalities using a 4-tier semiquantitative consensus scores**. (B) **Interobserver agreement between all three pathologists using a 4-tier semiquantitative scores for each modality**

| **Intraobserver agreement between modalities** | 4-tier scoring system | |
| --- | --- | --- |
|  | Kappa | Lower-Upper CI |
| SRH-H&E agreement | 0.55 | 0.45-0.66 |
| IHC-H&E agreement | 0.38 | 0.27-0.49 |
| IHC-SRH agreement | **0.69** | 0.59-0.79 |
| **Interobserver agreement between pathologists** | 4-tier scoring system | |
|  | Kappa | Lower-Upper CI |
| SRH agreement | 0.32 | 0.29-0.36 |
| H&E agreement | 0.44 | 0.41-0.47 |
| IHC agreement | **0.75** | 0.74-0.77 |

Kappa coefficients were measured for agreement between pathologists using 4-tiered scoring system. There was substantial agreement between SRH and IHC scores as illustrated by Κ 0.69 (CI 0.59-0.79). Agreement between IHC and H&E was fair with Κ 0.38 (CI 0.27-0.49), and agreement between SRH and H&E were moderate with Κ 0.55 (CI 0.45-0.66). Interobserver agreement between all three pathologists varied based on modality for the 4-tier scoring systems. IHC consensus samples showed substantial agreement as illustrated by, Κ 0.75 (CI 0.74-0.77) (**Table 2B**). Kappa score for H&E-stained sections was 0.44 (CI 0.41-0.47). Kappa score for SRH images was 0.32 (CI 0.29-0.36).

**Supplemental Table 3: Intraobserver agreement between modalities using 4-tier semiquantitative scores for each pathologist.**

|  | 4-tier | | |
| --- | --- | --- | --- |
|  | (Pathologist #1) | (Pathologist #2) | (Pathologist #3) |
| SRH-H&E agreement | 0.27 (0.16, 0.37) | 0.56 (0.46, 0.65) | 0.65 (0.56, 0.73) |
| IHC-H&E agreement | 0.52 (0.42, 0.63) | 0.80 ( 0.72, 0.88) | 0.53 (0.42, 0.63) |
| IHC-SRH agreement | 0.19 (0.08, 0.30) | 0.49 (0.39, 0.60) | 0.28 (0.17, 0.39) |

We then evaluated the intraobserver agreements between modalities for each individual pathologist. The agreements using 4-tier scoring system ranged from fair to almost perfect with significant variation based on the pathologists’ level of experience with SRH images.

**Supplementary Figure S1: Cell Counting Quantification Troubleshooting**

Cell counting quantification troubleshooting includes manual inspection for small samples in which white space covers a portion of the field of view area. Imaging autofocus (A) may result in counter failures therefore manual focus must be applied (B). Tumor margin specimen in which blood impacts imaging quality are manually excluded.

**
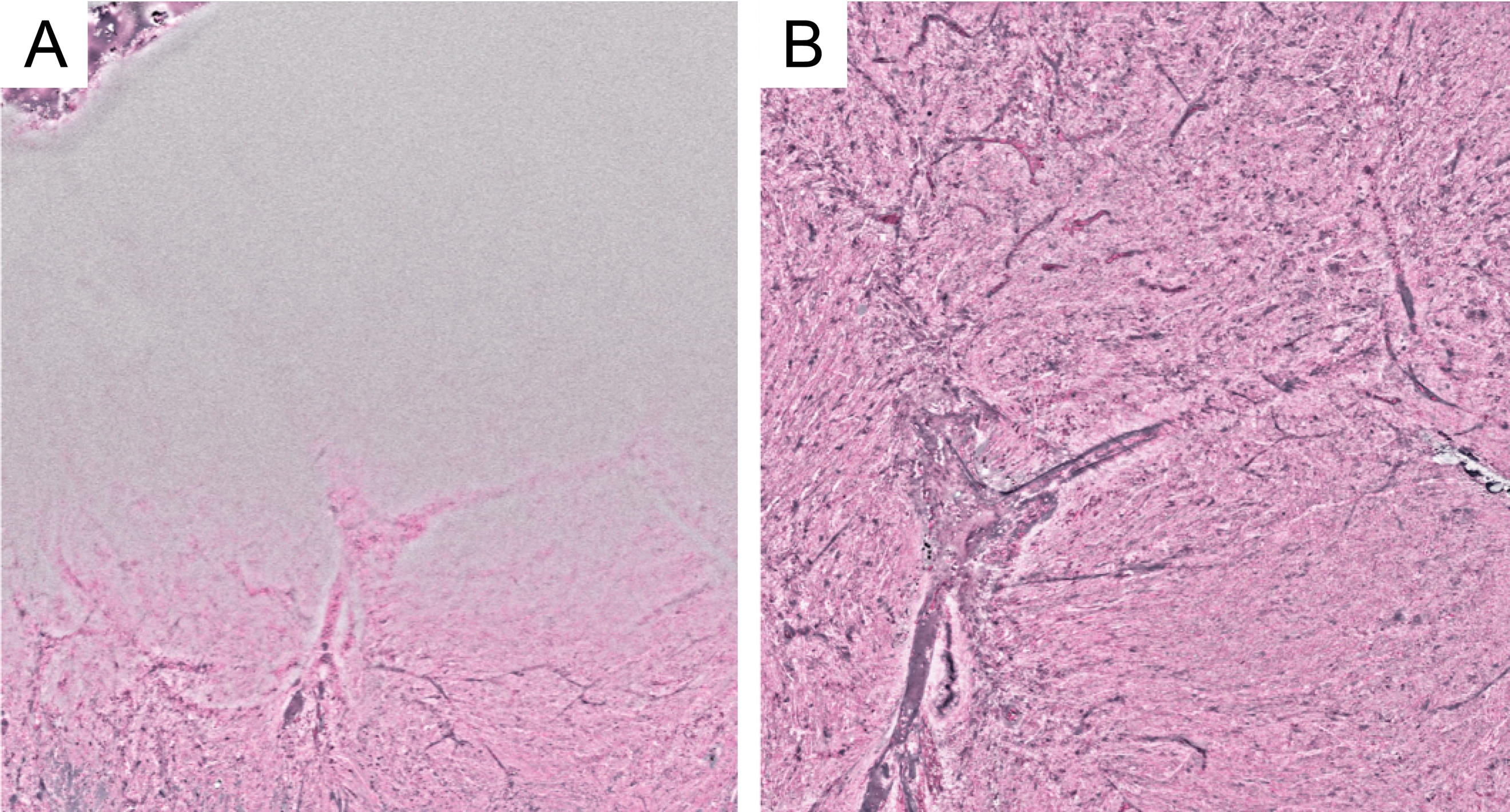
**

**Supplementary Figure S2: SRH Cellularity**

**(A)** Of all images with SRH consensus score n= 164, ROC curve 0-1 vs 2-3 area = 0.84. **(B)** Duplicates were identified and excluded so that final cell count analysis included n= 155 (9 excluded cases due to poor image quality). ROC and area under the curve was performed with duplicate filtering n = 155, ROC curve 0-1 vs 2-3 area = 0.85 and naïve cell-counts. This resulted in a modest improvement in performance after discarding samples with poor image quality and re-imaging these samples. However poor image samples remained given occasional small tissue samples in which uncovered slide and water make up a percentage of the image. **(C)** We then applied SRH operator estimates of good tissue percentage as each image was inspected and estimated for percentage of “good” tissue using a 10-point scale of 1-100 (100% = entire image is covered with tissue and 0= entire slide is non-tissue i.e. blood, plastic, water). Adjusting for this percentage, ROC curves improved, n = 155, ROC curve 0-1 vs 2-3 area = 0.86). The above approach is however reliant on human interpretation of percentage of good tissue coverage/ area. **(D)** In order to avoid potential human error we then applied a machine learning methodology to identify “white-space-areas” by a deterministic threshold (red is considered “excluded”). This approach does not allow the identification of blood which was interpretable using the operator inspection method. Machine leaning area under the curve therefore performed slightly worse than our manual operator inspection method due to the inability to interpret blood (n= 155, ROC curve 0-1 vs 2-3 area = 0.84). The AUC values support this hypothesis because the low-cellularity images would be more sensitive to bloody specimen (0 vs 1-2-3 AUC is worse than manual classification). Furthermore, the very high cellularity images (0-1-2 vs 3) would not be as sensitive to intermittent blood on the image and are actually improved (increased AUC).


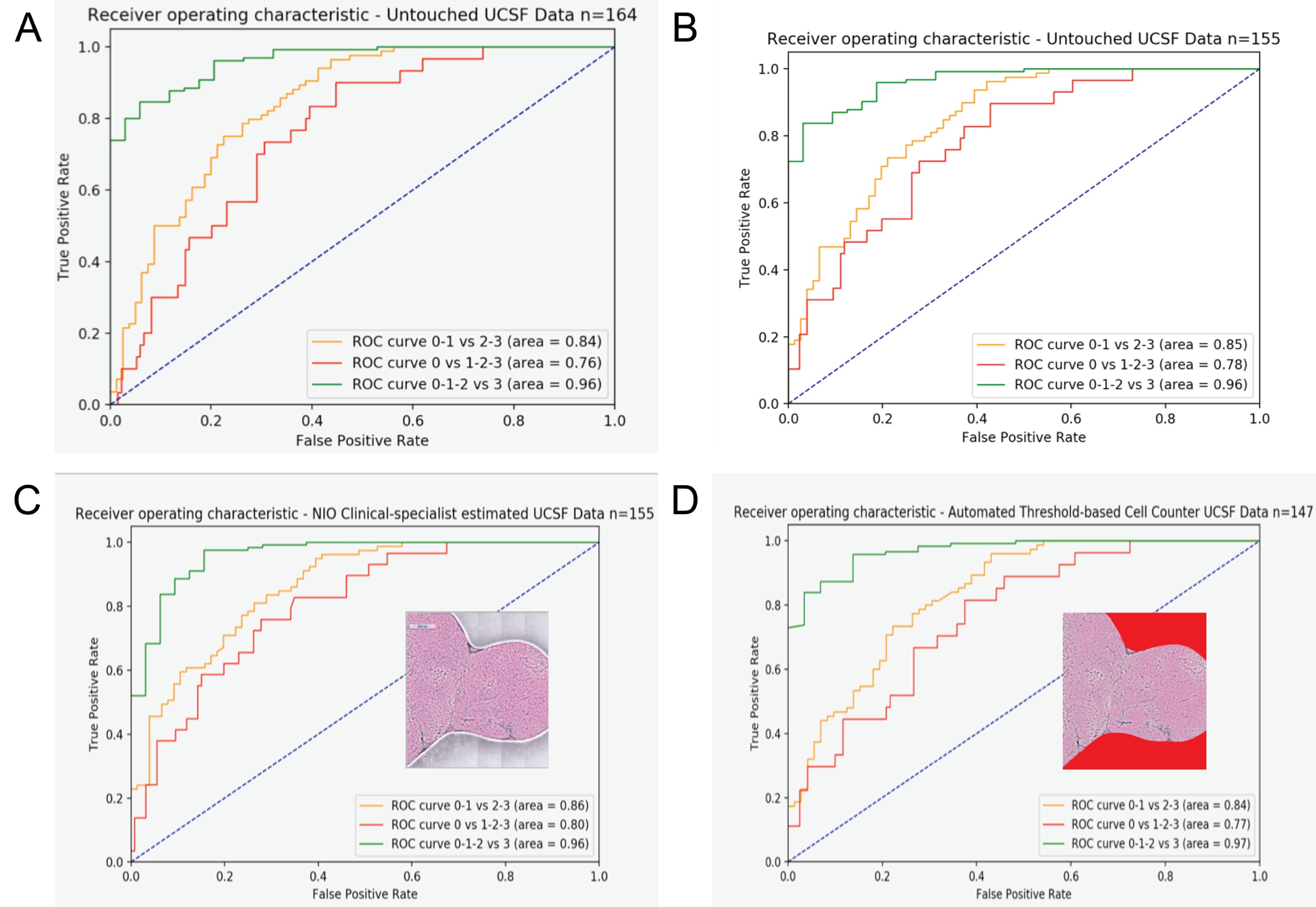

Supplement: Supplementary file 1 — Supplementary Information. [file 41598_2021_91648_MOESM1_ESM.docx]
